# The impact of pharmacist-led education and prospective audit and feedback on antibiotic dose optimization within medical intensive care units in Thailand: a retrospective study

**DOI:** 10.1080/20523211.2025.2467456

**Published:** 2025-02-28

**Authors:** Tipanong Gatechan, Chotirat Nakaranurack, Rongpong Plongla, Thanawan Chuenjit, Alan Edward Gross

**Affiliations:** a Clinical Pharmacy Unit, Department of Pharmacy, Sunprasitthiprasong Hospital, Ubon Ratchatani, Thailand; b Department of Pharmacy Practice, Faculty of Pharmaceutical Sciences, Chulalongkorn University, Bangkok, Thailand; c Division of Infectious Diseases, Department of Medicine, Faculty of Medicine, Chulalongkorn University and King Chulalongkorn Memorial Hospital, Thai Red Cross Society, Bangkok, Thailand; d Center of Excellence in Antimicrobial Resistance and Stewardship, Faculty of Medicine, Chulalongkorn University, Bangkok, Thailand; e Department of Clinical Pharmacy Unit, King Chulalongkorn Memorial Hospital, Thai Red Cross Society, Bangkok, Thailand; f Department of Pharmacy Practice, Retzky College of Pharmacy, University of Illinois Chicago, Chicago, IL, USA

**Keywords:** Pharmacist-led, education, prospective audit and feedback, antibiotic, dose optimization, medical intensive care units

## Abstract

**Background:**

Critical illness can affect antimicrobial pharmacokinetics and pharmacodynamics. Antimicrobial stewardship programs promote appropriate antimicrobial usage. This study aimed to compare the appropriateness of antibiotic dosing, therapeutic drug monitoring, and ICU mortality before and after antimicrobial stewardship program implementation in medical intensive care units.

**Methods:**

This retrospective study was conducted at King Chulalongkorn Memorial Hospital, Thailand. Adults admitted to medical intensive care units from August 1, 2019, to July 31, 2021, who received selected antibiotics in the antimicrobial stewardship program were included. During the intervention period, general education as well as prospective audit with intervention and feedback were implemented by infectious disease pharmacist and clinical pharmacists. The appropriateness of dosing, therapeutic drug monitoring, and ICU mortality were compared before and after antimicrobial stewardship program implementation.

**Results:**

There were 269 patients (455 prescriptions) and 376 patients (604 prescriptions) in the pre- and post-antimicrobial stewardship program implementation groups, respectively. Meropenem was the commonly prescribed antibiotic in both groups. Overall, the appropriateness of dosing and therapeutic drug monitoring improved after antimicrobial stewardship program implementation (36% to 63.58%, *p* < 0.001). Infectious disease and clinical pharmacists provided 40 interventions with an 87.5% acceptance rate. The most common recommendation was maintenance dose adjustment (79.17% acceptance rate). ICU mortality (29.37% to 18.62%, *p* = 0.001) and length of hospital stay in the ICU (7 days to 5 days, *p* = 0.005) were lower in the post-antimicrobial stewardship program implementation group.

**Conclusions:**

Pharmacist-led education and prospective audit and feedback on antibiotic dose optimization can improve appropriate antibiotic dosing and therapeutic drug monitoring with a high acceptance rate. We suggest implementing this strategy in other intensive care units such as surgical intensive care units. We still found some nonadherence to our dosing guidelines; additional strategies to optimize dosing should be evaluated.

## Background

In 2017, the estimated worldwide burden of sepsis was 48.9 million, with 11 million sepsis-related deaths (Rudd et al., 2020). Patients admitted to intensive care units (ICUs) are at high risk of progressing to sepsis and thus ICUs typically have the greatest consumption of antibiotics and greatest risk of being infected with multidrug-resistant (MDR) pathogens among all inpatient settings (Gotts & Matthay, 2016). Furthermore, infections due to MDR pathogens are associated with increased mortality, length of stay, and cost of treatment (Sunenshine et al., 2007; Thaden et al., 2017).

There was a high consumption of antibiotics in the ICU. A previous study in Malawi showed that 81.6% of patients in the ICU received antibiotics (Kayambankadzanja et al., 2020). Patient physiology and pharmacokinetic parameters, especially antibiotic change during sepsis and septic shock. For example, patients may be fluid-overloaded or hyperdynamic, and thus, drug concentrations may be decreased, especially for hydrophilic antibiotics (Varghese et al., 2011). Or patients may have diminished renal function and need renal replacement therapy (RRT), which impacts the exposure and dosing of renally-eliminated antibiotics (Roberts & Lipman, 2009; Udy et al., 2013). Therefore, ensuring optimal antibiotic dosing is especially important in critically ill patients. Underlining this, a recent study found that antibiotics were the leading cause of drug-related problems in the ICUs setting with 37.1% of those being related to dose selection (Li et al., 2020).

There was a statistically significant association between the inappropriate antibiotic therapy and high mortality among ICU patients (*p*-value = 0.014) (Abdelkarim et al., 2023). Antimicrobial Stewardship Programs (ASP) are one of the systematic approaches that can minimize the inappropriate use of antibiotics (Ture et al., 2023). The Infectious Diseases Society of America (IDSA), the Society for Healthcare Epidemiology of America (SHEA), and the Pediatric Infectious Diseases Society (PIDS) define ASP as ‘Coordinated interventions designed to improve and measure the appropriate use of [antibiotic] agents by promoting the selection of the optimal [antibiotic] drug regimen including dosing, duration of therapy, and route of administration’ (Barlam et al., 2016). ASP aims to improve patient outcomes, reduce adverse events such as *Clostridioides difficile* infection, minimize the development of antibiotic resistance, and optimize resource utilization across the continuum of care via a multidisciplinary team (Barlam et al., 2016). Data from previous systematic reviews showed that ASP can reduce antibiotic consumption and inappropriate prescribing. Additionally, some studies showed economic benefits from ASP (Abdel Hadi et al., 2024).

The 2016 IDSA guideline for implementing an antibiotic stewardship program recommends prospective audit and feedback as one of the two core strategies of effective ASP (Barlam et al., 2016). Implementing prospective audit and feedback in ICUs can increase the appropriate use of antibiotics, reduce MDR pathogens, decrease antibiotic use and antibiotic expenditures (Amer et al., 2013; DiazGranados, 2012; Morris et al., 2019; Taggart et al., 2015). For example, one study found that implementing an ASP in medical intensive care units (MICUs) significantly increased the matching to antibiotic policy from 76% to 89.33% (Shah et al., 2017).

There is only limited data reporting the outcomes of ASP in Thailand, especially ASP implementation in the ICUs setting. A survey assessing characteristics associated with ASP implementation in Thailand showed that hospitals with medical school affiliations (aOR = 46.47) and participation in a collaborative programs to prevent healthcare-associated infections (aOR = 41.14) were associated with ASP implementation (Khawcharoenporn et al., 2013). Another Thai study found that ASP with a pharmacist participating in the medical wards was associated with more appropriate antibiotic prescribing relative to ASP without a pharmacist (89.2%, and 78.2% respectively, *p* = 0.008) (Ananwattanakit et al., 2015).

The ASP at King Chulalongkorn Memorial Hospital (KCMH), Thailand, was started in 2012 and was focused in one medical ward. Since then, it has expanded to six medical wards, and eleven pediatric wards, and a multidisciplinary ASP committee was established in 2018. In 2020, the ASP activities were expanded to two MICUs. The first phase of implementation in our MICUs was mainly focused on optimizing antibiotic dosing, and therapeutic drug monitoring (TDM). This study aimed to compare the appropriateness of antibiotic dosing and TDM before and after ASP implementation in our MICUs. Additionally, we also assessed the ICU mortality rate, 30-day hospital mortality, and length of ICU stay before and after ASP implementation.

## Methods

This was a retrospective study conducted at KCMH, a 1,479-bed tertiary and teaching hospital in Bangkok, Thailand. The patient inclusion criteria were as follows: (1) ≥ 18 years of age who received care in MICUs from August 1, 2019 to July 31, 2021 and (2) Received one of the following intravenous antibiotics, including meropenem, imipenem/cilastatin, ertapenem, piperacillin/tazobactam, cefoperazone/sulbactam, ampicillin/sulbactam, sulbactam, colistin, fosfomycin, amikacin, gentamicin or vancomycin. Patients were excluded if their medical records were incomplete. The Institutional Review Board of the Faculty of Medicine, Chulalongkorn University, Bangkok, Thailand approved this study (COA no. 0059/2022).

The pre-ASP period was from August 1, 2019 to July 31, 2020 and the post-ASP period was from August 1, 2020 to July 31, 2021. In the intervention period, prospective audit and feedback by a rounding infectious disease (ID) pharmacist and 2 clinical pharmacists occurred twice weekly. If the ID pharmacist or clinical pharmacists identified inappropriately dosed or inadequately monitored (via TDM) prescriptions, they discussed it with the MICUs medical residents. Additionally, the ID pharmacist provided presentations on optimal antibiotic dosing and TDM in critically ill patients once monthly for medical residents. The ID pharmacist and clinical pharmacists also explained the criteria for appropriateness of antibiotic dosing and TDM to medical residents. Our ASP committee included ID physicians, ID pediatricians, an ID pharmacist, clinical pharmacists, chief nurses, microbiologists, and the information technology team. Our committee met every 3–4 months, and clinical pharmacists and the ID pharmacist presented baseline and outcomes data related to the ASP implemented in the MICUs. At the beginning of the implementation, ID physicians, the ID pharmacist, and the clinical pharmacists met with the assistant director of critical care and emergency unit, nurses, and the critical care committee to present our objectives, activities, and the program's evaluation criteria. We also presented our program to all medical residents at the orientation ceremony. The primary endpoint was the appropriateness of antibiotic dosing and TDM before and after ASP implementation. The secondary endpoints included physician acceptance rate of pharmacist interventions, antibiotic usage, ICU mortality, 30-day in-hospital mortality, time to extubation, and time to discontinuation of inotropic drugs before and after ASP implementation.

### Definitions

Each new prescription for the aforementioned antibiotics were included if they met one of the following criteria: (i) Dose change based on renal or hepatic function or changes in RRT. (ii) Dose change in response to TDM. (iii) Change to a new antibiotic based on susceptibility or clinical symptoms. The ID pharmacist and clinical pharmacists were responsible for evaluating the appropriateness of antibiotic dosing and TDM for each antibiotic.

Criteria for the appropriateness of antibiotic dosing included the use of an initial loading dose for colistin (300 mg colistin base activity) and vancomycin (20-35 mg/kg). Additionally, extended infusions should be used for maintenance doses of meropenem, imipenem/cilastatin, piperacillin/tazobactam, cefoperazone/sulbactam, ampicillin/sulbactam, sulbactam and fosfomycin. Upon initiation of these agents infused via extended infusion, the first dose should be infused over 30–60 mins to ensure rapid adequate serum concentrations. For example, patients who received meropenem should receive 1000 mg infused over 30 mins for the first dose, then 1000 mg infused over 3 hours every 8 hours. Finally, for drugs dosed by renal function, renal dose adjustment should be deferred for the first 24 hours of antibiotic initiation to ensure early adequate serum concentrations. This was approach was promoted knowing that renal function may improve or decline further after the first 24 hours and early adequate concentrations are essential in critically ill patients. After 24 hours on the antibiotic being initiated, the agent should then be dose adjusted by renal function and/or renal replacement therapy (RRT). For example, in patients with an estimated creatinine clearance (CrCl) of 30 mL/minute on day 1 and 2 of antibiotic initiation, they would receive meropenem 1000 mg every 8 hours for the first 24 hours (McKenzie, 2011) and then their dose would be renally-adjusted to 1000 mg every 12 hours thereafter (based on LEXIDRUG^®^).

Prescriptions were considered to have appropriate therapeutic drug monitoring if aminoglycosides and vancomycin dosing and TDM was conducted based on the LEXIDRUG^®^ application and clinical studies. The full evaluation criteria and referenced studies are presented in the Supplemental Tables S1–S5.

Secondary endpoints were defined as follows: ICU mortality rate was defined as the number of included patients who died in the MICUs from any cause divided by the number of all included patients in the study period. The 30-day hospital mortality rate was defined as the number of patients who died from any cause and occurred in the hospital within 30 days from the first day that patients received target antibiotics divided by the number of all patients during the in-study period. Time to extubation was defined as the duration since patients were initiated on mechanical ventilation and received targeted antibiotics in the MICUs until the patient was extubated within any hospital location. Time to discontinuation of inotropic drugs was defined as the duration since patients were initiated on inotropic drugs with targeted antibiotics at the MICUs until the patient stopped receiving inotropic drugs at any wards. Defined Daily Dose (DDD/1,000 patient-days) was defined as the average maintenance dose per day for a drug used for its main indication in adults, followed by World Health Organization (WHO) divided by 1,000 patient-days (Supplement) (Organization, 2020). We calculated DDD in each of the antibiotics monthly. Days of therapy (DOT/1,000 patient-days) were measured as one day when a patient is given a drug, regardless of dose, divided by 1,000 patient-days (Pakyz et al., 2009). We calculated DOT in each antibiotic monthly. Physicians’ acceptance was defined as the implementation of the pharmacists’ intervention by physicians.

Sepsis was defined as a systemic inflammatory response to a confirmed or suspected infection. Clinically, Systemic Inflammatory Response Syndrome (SIRS) is the occurrence of at least two of the following criteria: Temperature > 38.0°C or <36.0°C, heart rate >90 beats/minute respiratory rate >20 breaths/minute, white blood cells >12,000 cell/mm^3^ or < 4,000 cell/mm^3^. Septic shock was defined as sepsis in patients with a systolic blood pressure of ≤ 90 mmHg from the baseline despite adequate fluid resuscitation or those requiring vasopressor agents (Bone et al., 1992). The Acute Physiology and Chronic Health Evaluation II (APACHE II) score is a severity of illness and mortality estimation tool that was calculated as previously described (Knaus et al., 1985). Multidrug-resistant (MDR) was defined as a pathogen that was non-susceptible to ≥1 agent in ≥3 antimicrobial categories, extensively drug-resistant (XDR) was a pathogen that was non-susceptible to ≥1 agent in all but ≤2 antimicrobial categories, and pan-drug-resistant (PDR) was a pathogen that was non-susceptible to all antimicrobial agents listed (Magiorakos et al., 2012). For baseline characteristics, we reported the first episode of MICUs admission data for patients with multiple MICUs encounters, e.g., age, APACHE II score, comorbidity, and septic shock.

### Statistical analysis

Baseline characteristics and outcomes were compared before and after the implementation of the ASP. We assessed the normality of continuous data by Shapiro–Wilk normality test. Mean with standard deviation (±SD) and median with interquartile range (IQR) were used to describe continuous data. The independent t-test or Mann–Whitney U test was used for comparing continuous data. We will use the independent t-test for data that follows a normal distribution. Categorical data were expressed as frequencies and percentages. The Chi-square test or Fisher’s exact test assessed associations between categorical data. We will use Fisher’s exact test when our data shows more than 20% of cells have expected frequencies less than 5. Variables with a *P*-value < 0.05 were considered statistically significant. A logistic regression model was developed to identify factors associated with ICU mortality. Odds ratio (ORs) and associated 95% confidence interval (95% CI) were calculated. Variables with *P*-value < 0.1 in the univariate analysis were included in the multivariate analysis. Variables with a *P*-value < 0.05 were considered statistically significant in multivariate analysis. All statistical analysis data were performed using Statistical Package for the Social Science (SPSS) software for Windows, Version 22.0. (IBM corp., Armonk, NY).

## Results

Six hundred and sixty-two patients with 1078 antibiotic prescriptions were included in the study. We excluded 17 patients (19 prescriptions) due to incomplete medical records. Two hundred and sixty-nine patients (455 prescriptions) and 376 patients (604 prescriptions) were in the pre-ASP and post-ASP implementation groups, respectively. Most baseline characteristics were similar in both groups (Table 1). Pneumonia was the most common infection identified and meropenem was the most frequently used antibiotic in both groups. *Acinetobacter baumannii* was the most common pathogen in the pre-ASP period (23.74%), whereas *Klebsiella pneumoniae* was the most common pathogen (12.42%) in the post-ASP implementation.

**Table 1.** Baseline characteristic of patients in the Pre-ASP and Post-ASP implementation groups

**Characteristics**	**Pre-ASP**	**Post-ASP**	*P*-value
	*N* = 269	*N* = 376	
Male (*N*, %)	140 (52.04)	226 (60.11)	0.042*
Age (mean ± SD)	64.66 ± 19.20	66.39 ± 16.81	0.237
Comorbidity (*N*, %)	239 (88.85)	351 (93.35)	0.043*
Cardiovascular disease^a^	160 (59.48)	230 (61.17)	0.665
Metabolic syndrome^b^	120 (44.61)	195 (51.86)	0.079
Cancer	60 (22.30)	110 (29.26)	0.051
Renal impairment^c^	53 (19.70)	84 (22.34)	0.419
Immunocompromised host^d^	17 (6.32)	23 (6.12)	0.916
Pulmonary disease^e^	46 (17.10)	62 (16.49)	0.838
APACHE II score (mean ± SD)	19.20 ± 6.39	19.90 ± 6.05	0.066
Septic shock	111 (41.26)	164 (43.62)	0.551
Post COVID-19 infection^f^	1 (0.37)	18 (4.79)	0.001*
Consult ID physician (*N*, %)	147 (54.65)	190 (50.53)	0.302
**Source of infection (*N*, %)**			
Pneumonia	163 (60.59)	189 (50.27)	0.009*
Bloodstream infection	68 (25.28)	90 (23.94)	0.696
Skin and soft tissue infection	26 (9.67)	28 (7.45)	0.316
Intra-abdominal infection	19 (7.06)	27 (7.18)	0.954
Unknown sources of infection	30 (11.15)	62 (16.49)	0.056
**Pathogens (*N*, %)^†^**			
*Acinetobacter baumannii*	108 (23.74)	54 (8.94)	<0.001*
*Klebsiella pneumoniae*	62 (13.63)	75 (12.42)	0.562
*Pseudomonas aeruginosa*	51 (11.21)	61 (10.10)	0.561
*Escherichia coli*	47 (10.33)	72 (11.92)	0.471
*Staphylococcus aureus*	10 (2.20)	16 (2.65)	0.639
**Culture-negative (*N*,%)^†^**	166 (36.48)	267 (44.21)	0.011*
**Resistant pathogens (*N*, %)^†^**			
Muti-drug resistance (MDR)	83 (18.24)	158 (26.16)	0.002*
Extensively drug resistance (XDR)	80 (17.58)	89 (14.74)	0.210
Pan-drug resistance (PDR)	97 (21.32)	63 (10.43)	<0.001*
**Type of antibiotics (*N*, %)^†^**			
Meropenem	268 (58.90)	398 (65.89)	0.025*
Piperacillin/tazobactam	128 (28.13)	145 (24.01)	0.129
Colistin	104 (22.86)	72 (11.92)	<0.001*
Vancomycin	58 (12.75)	105 (17.38)	0.038*
Cefoperazone/sulbactam	28 (6.15)	13 (2.15)	0.001*
Ampicillin/sulbactam	20 (4.40)	6 (0.99)	<0.001*
Sulbactam	23 (5.05)	17 (2.81)	0.057
Fosfomycin	25 (5.49)	11 (1.82)	0.001*
Imipenem/cilastatin	14 (3.08)	4 (0.66)	0.003*
Ertapenem	3 (0.66)	3 (0.50)	1.000
Amikacin	3 (0.66)	3 (0.50)	1.000
Gentamicin	3 (0.66)	3 (0.50)	1.000
Combination therapy	135 (29.67)	123 (20.36)	<0.001*

^†^ The value calculated from the amount of prescriptions in each period (pre-AS*P* = 455 prescriptions and post-AS*P* = 604 prescriptions).

*Chi-square test.

^a^ Cardiovascular disease: Hypertension, Ischemic heart disease, Stroke, Atrial fibrillation, Heart failure, Deep vein thrombosis.

^b^ Metabolic syndrome: Dyslipidemia, Diabetes mellitus, Thyroid disorder.

^c^ Renal impairment: Chronic kidney disease, End-stage renal disease.

^d^ Immunocompromised host: HIV, Transplant organ host, Patient received immunosuppressive drugs.

^e^ Pulmonary disease: Asthma, Pulmonary tuberculosis infection, Chronic obstructive pulmonary disease.

^f^ COVID-19: coronavirus disease 2019.

**Figure 1.**
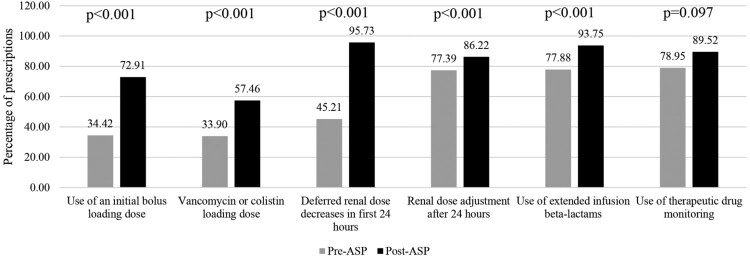
Appropriateness of dosing, administration and therapeutic drug monitoring before and after ASP implementation.

**Figure 2.**
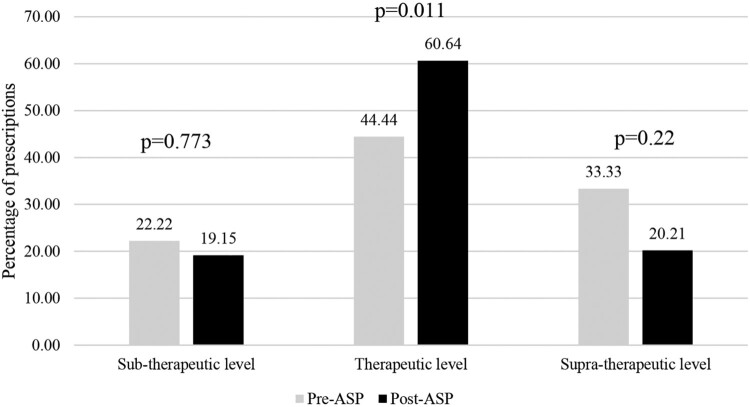
Percentage of prescriptions attaining initial therapeutic concentrations for aminoglycosides and vancomycin

Appropriately dosed and TDM-ordered prescription improved after implementation of the ASP intervention, from 36% to 63.58% (*p* < 0.001). Our study found a statistically significantly increased proportion of appropriate dosing, administration, and therapeutic drug monitoring for most aspects (Fig. 1). The proportion of therapeutic drug monitoring increased, but the change was not statistically significant (*p* = 0.097). However, more patients achieved an initial therapeutic level after the ASP intervention, increasing from 44.44% to 60.64% (*p* = 0.011) (Figure 2).

After implementation of the ASP, pharmacists intervened 40 times via prospective audit, with an intervention acceptance rate of 87.5% (Table 2). The most frequent intervention was a maintenance dose adjustment. The five instances where physicians did not accept the pharmacists’ intervention were as follows: Dose increase recommended based on CrCl or RRT [3 times, 3 patients]; Dose increase recommended based on RRT and the minimum inhibitory concentration of the pathogen [2 times, 1 patient].

**Table 2.** Type of pharmacists’ interventions and physicians’ acceptance rate post-ASP implementation

**Type of intervention**	**No. of interventions**	**No. accepted (%)**
Maintenance dose adjustment	24	19 (79.17)
Recommend TDM	8	8 (100)
Recommend extended infusion	5	5 (100)
Dose adjustment after TDM	3	3 (100)
**Total**	40	35 (87.5)

TDM = therapeutic drug monitoring.

Table 3 presents the secondary clinical outcomes in the pre- and post-ASP groups. There was a lower ICU mortality rate in the post-ASP implementation compared to the pre-ASP period (18.62% vs 29.37%, *p* = 0.001) and a decreased length of stay in ICU. However, there were no significant differences in 30-day hospital mortality, time to extubation, and time to resolution of septic shock.

**Table 3.** Clinical outcome of patients in the Pre-ASP and Post-ASP implementation at MICUs, KCMH from August 1, 2019, to July 31, 2021

**Clinical outcome**	**Pre-ASP** *N* = 269	**Post-ASP** *N* = 376	*P*-value
ICU mortality (N, %)	79 (29.37)	70 (18.62)	0.001*
The 30-day hospital mortality (N, %)	105 (39.03)	142 (37.77)	0.772
Time to off ventilator (median, IQR) days	8 (4,17)	8 (4,14)	0.239
Time to resolution of septic shock (median, IQR) days	4 (2,7)	4 (3,7)	0.358
Length of stay in ICU (median, IQR) days	7 (4, 14.50)	5 (3,11)	0.005**

ICU = Intensive care unit, IQR = interquartile range, *Chi-square test, **Mann-Whitney U test.

There were no changes in the median antibiotic DDD/1,000 patient-days pre and post-ASP implementation (2,562.60 VS 2,196.58, *p* = 0.525) and DOT/1,000 patient-days (2,542.64 VS 1,761.46, *p* = 0.204) (see additional details in Supplemental Material).

Multivariate analysis showed factors associated with ICU mortality were APACHE II score (aOR = 1.04, *P*-value = 0.015), septic shock (aOR = 2.86, *P*-value < 0.001), and received CRRT (aOR = 2.59, *P*-value <0.001). Post-ASP implementation (aOR = 0.56, *P*-value = 0.005) and XDR pathogen infection (aOR =   0.50, *P*-value  = 0.038) were found to be protective factors. The data are shown in Table 4.

**Table 4.** Factors associated with ICU mortality of all patients in the Pre-ASP and Post-ASP implementation

	**Univariate analysis**			**Multivariate analysis**		
	**OR**	**95%CI**	*P*-value	**aOR**	**95%CI**	*P*-value
**Male**	0.95	0.66–1.38	0.78			
**Age**	1.00	0.991–1.012	0.74			
**APACHE II score**	1.07	1.04–1.11	<0.001*	1.04	1.01–1.08	0.015**
**Comorbidity**	0.87	0.46–1.64	0.67			
**Immunocompromise host**	0.68	0.34–1.38	0.29			
**Post-ASP implementation**	0.55	0.38–0.80	0.002*	0.56	0.37–0.84	0.005**
**Pathogen**						
*Acinetobacter baumannii*	2.39	1.42–4.04	0.001*			N.S.
*Klebsiella pneumoniae*	1.25	0.69–2.28	0.46			
**Pattern resistant pathogens**						
MDR	1.02	0.67–1.57	0.91			
XDR	0.55	0.30–0.99	0.05*	0.50	0.26–0.96	0.038**
PDR	2.523	1.49–4.28	0.001*			N.S.
**Source of infection**						
Pneumonia	1.27	0.88–1.83	0.21			
Bloodstream infection	1.60	1.07–2.40	0.023*			N.S.
Unknown sources of infection	0.36	0.18–0.72	0.004*			N.S.
** Septic shock**	3.21	2.19–4.70	<0.001*	2.86	1.84–4.42	<0.001**
** Post COVID–19 infection**	1.20	0.42–3.38	0.74			
**Received ventilator**	2.81	1.53–5.16	0.001*			N.S.
**Renal replacement therapy**						
CRRT	3.59	2.24–5.76	<0.001*	2.59	1.52–4.42	<0.001**
**Amount of antibiotics per prescription**						
Monotherapy	0.42	0.27–0.66	<0.001*			N.S.
Combination therapy	2.39	1.53–3.75	<0.001*			N.S.

*Univariate analysis, **Multivariate analysis, N.S.: Not significant, OR = Odds ratio, aOR = adjusted Odds ratio, CI = Confidence interval, ASP = antimicrobial stewardship program, MDR = multidrug – resistant, XDR = extensivelydrug – resistant, PDR = Pan-drug-resistant, CRRT = continuous renal replacement therapy, APACHE II score = Acute Physiology and Chronic Health Evaluation II.

## Discussion

Our study found that implementing pharmacist-led antibiotic dose optimization via education and prospective audit and feedback in MICUs significantly improved the appropriateness of antibiotic dosing and TDM. We also found more patients achieved therapeutic vancomycin and aminoglycoside serum concentrations in the post-ASP implementation group. We chose to focus on antibiotic dosing and TDM optimization for the first phase of our MICUs ASP implementation given the physiologic and pharmacokinetic changes that occur during critical illness, which can affect patients’ drug exposure and clinical outcomes (Abdul-Aziz et al., 2020). Furthermore, before implementing our intervention, we noted anecdotally that there was significant opportunity to optimize antibiotic dosing in our MICU patients. Similar to our experience, a retrospective study of critically ill patients found that antibiotics were the leading medication class associated with drug-related problems (DRPs) (59.7%), and the leading problem was inappropriate antibiotic dosing (37.1%)(Li et al., 2020). Interventions similar to ours can impact patient outcomes; a study by Jiang et al. found that pharmacists participating in antimicrobial dosing adjustments in septic patients receiving CRRT was associated with a reduced ICU stay and fewer adverse drug events (Jiang et al., 2013).

A few previous studies conducted in Asia have shown that pharmacist-driven ASP can optimize antimicrobial use through different interventions (Apisarnthanarak et al., 2015; Fukuda et al., 2021; K. Jantarathaneewat et al., 2021; Kittiya Jantarathaneewat et al., 2022; Nakamura et al., 2021). In our pharmacist-led ASP, both the ID pharmacist and clinical pharmacists evaluated the appropriateness of antibiotic dosing and TDM via prospective audit and feedback twice a week. In addition, the ID pharmacist provided presentations on optimal antibiotic dosing and therapeutic drug monitoring in critically ill patients once monthly for medical residents. According to the IDSA guidelines for implementing an Antibiotic Stewardship Program 2016, they suggest against relying solely on didactic educational materials for stewardship (Barlam et al., 2016). The IDSA guidelines suggest the use of multiple strategies (e.g., antibiotic time-out, stop orders, prospective audit and feedback, and clinical practice guidelines) to increase the appropriateness of antibiotics regimens. It is well-documented that providing education with feedback can enhance compliance with the protocol or suggestion (Baccolini et al., 2019; Ebben et al., 2018). Therefore, we implemented prospective audit and feedback in addition to general educational strategies, and as a result improved the appropriateness of our antibiotic dosing and TDM. Although we found significant improvements in dosing and TDM after implementing our intervention, there is still further opportunity to optimize dosing. Other strategies, e.g., standing or preprinted orders and having full-time pharmacists at MICUs, might help further dosing in our MICUs. Furthermore, we may be able to use the results of our study to help justify additional pharmacist resources in the MICU setting to provide coverage of the service more than two days per week. Pharmacists in our study provided 40 interventions during the post-ASP implementation period, and maintenance dose adjustment was the most common intervention. The physicians’ acceptance rate in our study was as high as 87.5%. This high acceptance rate is similar to another ICU-based study evaluating prospective audit and feedback by an ASP that found a 78.4% acceptance rate overall and an 87% acceptance rate related to dose optimization interventions (Khdour et al., 2018).

Our study showed that the antibiotic dosing and TDM optimization strategies implemented in our MICUs were not associated with worse outcomes and in fact we identified a decrease in ICU mortality after implementation. Our study also found that ASP implementation was a protective factor for ICU mortality in multivariate analysis. There is biologic plausibility for this finding, specifically that our interventions aimed to facilitate rapid adequate antibiotic exposures in our most critically ill patients which may improve their clinical outcome. However, this outcome must be placed in context of our study design and the timing of our study. First, this was a non-randomized study and there are likely unmeasured confounders that impacted this outcome. Furthermore, the post-intervention period occurred exclusively after the start of the COVID-19 pandemic which likely impacted patient baseline severity of illness, type of microorganisms, and other characteristics during the post-intervention period. In contrast to our study, Ruiz and colleagues found that there was no change in all-cause ICU mortality (Ruiz et al., 2018). Another prospective observational cohort study by Aldardeer NF et al. showed that deferring renal dose adjustments in the first 24 hours of sepsis recognition were associated with a significant reduction in in-hospital mortality [HR = 0.588; 95% CI = 0.355-0.974] (Aldardeer et al., 2024). Finally, our study also found shorter lengths of stay in the ICU in the post-ASP period, which was similar to the study by Jiang et al (Jiang et al., 2013). Based on our study and the results of others, as well as the importance of achieving appropriate antibiotic exposures in critically ill patients, we expect dose optimization may improve patient outcomes and at least does not harm patients.

From our study, pharmacist-led education and prospective audit and feedback on antibiotic dosing and TDM optimization are safe and have a high acceptance rate. We advocate for the implementation of both strategies in critical care settings to the extent that resources allow. Notably, in August 2023, the US CDC has recommended that pharmacists provide direct patient care in ICU settings to facilitate administration of antibiotics to patients with sepsis (CDC, 2023). For the next phase of our MICUs ASP, we plan on implementing additional strategies to improve the appropriate choice and duration of antibiotics. Our study has some strengths and limitations. This is the first study in Thailand that evaluated the clinical outcome and antibiotic usage of pharmacist-led education and prospective audit and feedback on antibiotic dosing and TDM optimization at MICUs. This study has some limitations. Firstly, our study was retrospective, we couldn’t control for misclassification bias and unmeasured confounders. Secondly, our strategies focused on only antibiotic dosing and therapeutic drug monitoring optimization resulting in our result cannot representing the whole effects of ASP at MICUs. Thirdly, our study only evaluated some antibiotics, all of which are intravenous antibiotics, so we cannot apply our results to all antibiotics. Further, we didn’t collect data to characterize time to active therapy, which can affect patients’ outcomes. Lastly, the covid pandemic might affect the patients’ characteristics and clinical outcome as mentioned earlier.

## Conclusion

We demonstrated the successful implementation and outcomes of a pharmacist-led, multidisciplinary ASP in MICUs in our hospital in Thailand. We demonstrated an improvement in the proportion of patients receiving optimal antibiotic dosing and TDM. Our intervention was well-accepted, safe, and we were able to implement it even though prospective audit with intervention and feedback did not occur every day of the week. We hope these data and the evaluation of our program can support further expansion of our ASP in our ICUs and serve as a potential template for others looking to implement ASP in resource-constrained settings.

## Supplementary Material

Supplemental Material

## Data Availability

The datasets that were used and analyzed during this study are not publicly available due to confidentiality reasons. This restriction is imposed by the Med Chula Institutional Review Board. Data requests can be sent to corresponding author: chotirat.n@pharm.chula.ac.th.

## References

[CIT0001] Abdel Hadi, H., Eltayeb, F., Al Balushi, S., Daghfal, J., Ahmed, F., & Mateus, C. (2024). Evaluation of hospital antimicrobial stewardship programs: Implementation, process, impact, and outcomes, review of systematic reviews. *Antibiotics*, *13*(3), 253. 38534688 10.3390/antibiotics13030253 PMC10967423

[CIT0002] Abdelkarim, O. A., Abubakar, U., Taha, L. O., Ashour, S. A., Abass, W. Y., Osman, E. M., & Muslih, M. S. (2023). Impact of irrational use of antibiotics Among patients in the intensive care unit on clinical outcomes in Sudan. *Infection and Drug Resistance*, 7209–7217. 38023395 10.2147/IDR.S378645 PMC10656842

[CIT0003] Abdul-Aziz, M. H., Alffenaar, J.-W. C., Bassetti, M., Bracht, H., Dimopoulos, G., Marriott, D., Neely, M. N., Paiva, J.-A., Pea, F., Sjovall, F., Timsit, J. F., Udy, A. A., Wicha, S. G., Zeitlinger, M., De Waele, J. J. Roberts, J. A., & the Infection Section of European Society of Intensive Care, M., Pharmacokinetic/pharmacodynamic, Critically Ill Patient Study Groups of European Society of Clinical, M., Infectious, D., Infectious Diseases Group of International Association of Therapeutic Drug, M., Clinical, T., Infections in the, I. C. U., & Sepsis Working Group of International Society of Antimicrobial, C. (2020). Antimicrobial therapeutic drug monitoring in critically ill adult patients: A position paper#. *Intensive Care Medicine*, *46*(6), 1127–1153. 10.1007/s00134-020-06050-1 32383061 PMC7223855

[CIT0004] Aldardeer, N. F., Alshreef, M. M., Alharbi, E. A., Aljabri, A. K., Aljawadi, M. H., Almangour, T. A., Alobaili, S., Alarifi, M. I., Alomari, A., & Alhammad, A. M. (2024). Early versus late antipseudomonal β-lactam antibiotic dose adjustment in critically Ill sepsis patients With acute kidney injury: A prospective observational cohort study. *Open Forum Infectious Diseases*, *11*(3), 10.1093/ofid/ofae059 PMC10906704 38434610

[CIT0005] Amer, M. R., Akhras, N. S., Mahmood, W. A., & Al-Jazairi, A. S. (2013). Antimicrobial stewardship program implementation in a medical intensive care unit at a tertiary care hospital in Saudi Arabia. *Annals of Saudi Medicine*, *33*(6), 547–554. 24413857 10.5144/0256-4947.2013.547 PMC6074906

[CIT0006] Apisarnthanarak, A., Lapcharoen, P., Vanichkul, P., Srisaeng-Ngoen, T., & Mundy, L. M. (2015). Design and analysis of a pharmacist-enhanced antimicrobial stewardship program in Thailand. *American Journal of Infection Control*, *43*(9), 956–959. 10.1016/j.ajic.2015.05.011 26095656

[CIT0007] Baccolini, V., D’Egidio, V., de Soccio, P., Migliara, G., Massimi, A., Alessandri, F., Tellan, G., Marzuillo, C., De Vito, C., Ranieri, M. V., & Villari, P. (2019). Effectiveness over time of a multimodal intervention to improve compliance with standard hygiene precautions in an intensive care unit of a large teaching hospital. *Antimicrobial Resistance & Infection Control*, *8*(1), 92. 10.1186/s13756-019-0544-0 31164981 PMC6544958

[CIT0008] Barlam, T. F., Cosgrove, S. E., Abbo, L. M., MacDougall, C., Schuetz, A. N., Septimus, E. J., Srinivasan, A., Dellit, T. H., Falck-Ytter, Y. T., Fishman, N. O., Hamilton, C. W., Jenkins, T. C., Lipsett, P. A., Malani, P. N., May, L. S., Moran, G. J., Neuhauser, M. M., Newland, J. G., Ohl, C. A., … Trivedi, K. K. (2016). Implementing an antibiotic stewardship program. Guidelines by the Infectious Diseases Society of America and the society for healthcare epidemiology of America. *Clinical Infectious Diseases*, *62*(10), e51–e77. 10.1093/cid/ciw118 27080992 PMC5006285

[CIT0009] Bone, R. C., Balk, R. A., Cerra, F. B., Dellinger, R. P., Fein, A. M., Knaus, W. A., Schein, R. M., & Sibbald, W. J. (1992). Definitions for sepsis and organ failure and guidelines for the use of innovative therapies in sepsis. *Chest*, *101*(6), 1644–1655. 1303622 10.1378/chest.101.6.1644

[CIT0010] CDC. (2023). Hospital Sepsis Program Core Elements https://www.cdc.gov/sepsis/pdfs/sepsis-core-elements-H.pdf. 10.1001/jama.2023.16693 PMC10877561 37616213

[CIT0011] DiazGranados, C. A. (2012). Prospective audit for antimicrobial stewardship in intensive care: Impact on resistance and clinical outcomes. *American Journal of Infection Control*, *40*(6), 526–529. 21937145 10.1016/j.ajic.2011.07.011

[CIT0012] Ebben, R. H. A., Siqeca, F., Madsen, U. R., Vloet, L. C. M., & van Achterberg, T. (2018). Effectiveness of implementation strategies for the improvement of guideline and protocol adherence in emergency care: A systematic review. *BMJ Open*, *8*(11), e017572. 10.1136/bmjopen-2017-017572 PMC6254419 30478101

[CIT0013] Fukuda, T., Tanuma, K., Iio, S., Saito, J., Komura, M., & Yamatani, A. (2021). Impact of a pharmacist-led antimicrobial stewardship program on the number of days of antimicrobial therapy for uncomplicated gram-negative bacteremia in a community hospital. *Cureus*, *13*(4), e14635. 10.7759/cureus.14635 34046272 PMC8140741

[CIT0014] Gotts, J. E., & Matthay, M. A. (2016). Sepsis: Pathophysiology and clinical management. *Bmj*, *353*. 10.1136/bmj.i1585 27217054

[CIT0015] Jantarathaneewat, K., Apisarnthanarak, A., Limvorapitak, W., Weber, D. J., & Montakantikul, P. (2021). Pharmacist-driven antibiotic stewardship program in febrile neutropenic patients: A single site prospective study in Thailand. *Antibiotics (Basel)*, *10*(4), 10.3390/antibiotics10040456 PMC8072986 33920541

[CIT0016] Jantarathaneewat, K., Camins, B., & Apisarnthanarak, A. (2022). The role of the clinical pharmacist in antimicrobial stewardship in Asia: A review. *Antimicrobial Stewardship & Healthcare Epidemiology*, *2*(1), e176. 36386007 10.1017/ash.2022.310 PMC9641507

[CIT0017] Jiang, S.-P., Zhu, Z.-Y., Ma, K.-F., Zheng, X., & Lu, X.-Y. (2013). Impact of pharmacist antimicrobial dosing adjustments in septic patients on continuous renal replacement therapy in an intensive care unit. *Scandinavian Journal of Infectious Diseases*, *45*(12), 891–899. 24024759 10.3109/00365548.2013.827338

[CIT0018] Kayambankadzanja, R. K., Lihaka, M., Barratt-Due, A., Kachingwe, M., Kumwenda, W., Lester, R., Bilima, S., Eriksen, J., & Baker, T. (2020). The use of antibiotics in the intensive care unit of a tertiary hospital in Malawi. *BMC Infectious Diseases*, *20*(1), 776. 10.1186/s12879-020-05505-6 33076857 PMC7574463

[CIT0019] Khawcharoenporn, T., Apisarnthanarak, A., & Mundy, L. M. (2013). National survey of antimicrobial stewardship programs in Thailand. *American Journal of Infection Control*, *41*(1), 86–88. 22727247 10.1016/j.ajic.2012.01.032

[CIT0020] Khdour, M. R., Hallak, H. O., Aldeyab, M. A., Nasif, M. A., Khalili, A. M., Dallashi, A. A., Khofash, M. B., & Scott, M. G. (2018). Impact of antimicrobial stewardship programme on hospitalized patients at the intensive care unit: A prospective audit and feedback study. *British Journal of Clinical Pharmacology*, *84*(4), 708–715. 29236303 10.1111/bcp.13486 PMC5867097

[CIT0021] Knaus, W. A., Draper, E. A., Wagner, D. P., & Zimmerman, J. E. (1985). APACHE II: A severity of disease classification system. *Critical Care Medicine*, *13*(10), 818–829. 3928249

[CIT0022] Li, X.-x., Zheng, S.-q., Gu, J.-h., Huang, T., Liu, F., Ge, Q.-g., Liu, B., Li, C., Yi, M., Qin, Y.-f., Zhao, R.-s., & Shi, L.-w. (2020). Drug-Related problems identified during pharmacy intervention and consultation: Implementation of an intensive care unit pharmaceutical care model. *Frontiers in Pharmacology*, *11*(1417), 10.3389/fphar.2020.571906 PMC7516263 33013415

[CIT0023] Magiorakos, A.-P., Srinivasan, A., Carey, R., Carmeli, Y., Falagas, M., Giske, C., Harbarth, S., Hindler, J., Kahlmeter, G., & Olsson-Liljequist, B. (2012). Multidrug-resistant, extensively drug-resistant and pandrug-resistant bacteria: An international expert proposal for interim standard definitions for acquired resistance. *Clinical Microbiology and Infection*, *18*(3), 268–281. 21793988 10.1111/j.1469-0691.2011.03570.x

[CIT0024] McKenzie, C. (2011). Antibiotic dosing in critical illness. *Journal of Antimicrobial Chemotherapy*, *66*(suppl_2), ii25–ii31. 10.1093/jac/dkq516 21398304

[CIT0025] Morakot Ananwattanakit, S. U., Tantawichien, T., Puttilerpong, C., & Pengsuparp, T. (2015). Effects of pharmacist participation in an antimicrobial stewardship program on appropriate antibiotic use. *Thai Pharmaceutical and Health Science Journal*, *10*(1), 1–9.

[CIT0026] Morris, A. M., Bai, A., Burry, L., Dresser, L. D., Ferguson, N. D., Lapinsky, S. E., Lazar, N. M., McIntyre, M., Matelski, J., & Minnema, B. (2019). Long-term effects of phased implementation of antimicrobial stewardship in academic ICUs: 2007–2015. *Read Online: Critical Care Medicine| Society of Critical Care Medicine*, *47*(2), 159–166. 10.1097/CCM.0000000000003514 30407951

[CIT0027] Nakamura, S., Arima, T., Tashiro, R., Yasumizu, S., Aikou, H., Watanabe, E., Nakashima, T., Nagatomo, Y., Kakimoto, I., & Motoya, T. (2021). Impact of an antimicrobial stewardship in a 126-bed community hospital with close communication between pharmacists working on post-prescription audit, ward pharmacists, and the antimicrobial stewardship team. *Journal of Pharmaceutical Health Care and Sciences*, *7*(1), 25. 10.1186/s40780-021-00206-x 34332639 PMC8325832

[CIT0028] Organization, W. H. (2020). Defined daily dose (DDD). Retrieved 27 November from https://www.who.int/toolkits/atc-ddd-toolkit/about-ddd.

[CIT0029] Pakyz, A. L., Gurgle, H. E., Ibrahim, O. M., Oinonen, M. J., & Polk, R. E. (2009). Trends in antibacterial use in hospitalized pediatric patients in United States academic health centers. *Infection Control & Hospital Epidemiology*, *30*(6), 600–603. 19419328 10.1086/597545

[CIT0030] Roberts, J. A., & Lipman, J. (2009). Pharmacokinetic issues for antibiotics in the critically ill patient. *Critical Care Medicine*, *37*(3), 840–851. 19237886 10.1097/CCM.0b013e3181961bff

[CIT0031] Rudd, K. E., Johnson, S. C., Agesa, K. M., Shackelford, K. A., Tsoi, D., Kievlan, D. R., Colombara, D. V., Ikuta, K. S., Kissoon, N., & Finfer, S. (2020). Global, regional, and national sepsis incidence and mortality, 1990–2017: Analysis for the Global Burden of Disease Study. *The Lancet*, *395*(10219), 200–211. 10.1016/S0140-6736(19)32989-7 PMC6970225 31954465

[CIT0032] Ruiz, J., Ramirez, P., Gordon, M., Villarreal, E., Frasquet, J., Poveda-Andres, J., Salavert-Lletí, M., & Catellanos, A. (2018). Antimicrobial stewardship programme in critical care medicine: A prospective interventional study. *Medicina Intensiva*, *42*(5), 266–273. 28882325 10.1016/j.medin.2017.07.002

[CIT0033] Shah, N., Joshi, A., & Ganguly, B. (2017). Impact of antibiotic stewardship program on prescribing pattern of antimicrobials in patients of medical intensive care unit. *Journal of Clinical and Diagnostic Research: JCDR*, *11*(7), FC11. 28892925 10.7860/JCDR/2017/27171.10237 PMC5583932

[CIT0034] Sunenshine, R. H., Wright, M.-O., Maragakis, L. L., Harris, A. D., Song, X., Hebden, J., Cosgrove, S. E., Anderson, A., Carnell, J., Jernigan, D. B., Kleinbaum, D. G., Perl, T. M., Standiford, H. C., & Srinivasan, A. (2007). Multidrug-resistant Acinetobacter infection mortality rate and length of hospitalization. *Emerging Infectious Diseases*, *13*(1), 97–103. 10.3201/eid1301.060716 17370521 PMC2725827

[CIT0035] Taggart, L. R., Leung, E., Muller, M. P., Matukas, L. M., & Daneman, N. (2015). Differential outcome of an antimicrobial stewardship audit and feedback program in two intensive care units: A controlled interrupted time series study. *BMC Infectious Diseases*, *15*(1), 480. 26511839 10.1186/s12879-015-1223-2 PMC4625716

[CIT0036] Thaden, J. T., Li, Y., Ruffin, F., Maskarinec, S. A., Hill-Rorie, J. M., Wanda, L. C., Reed, S. D., & Fowler, Jr V. G. (2017). Increased costs associated with bloodstream infections caused by multidrug-resistant gram-negative bacteria are due primarily to patients with hospital-acquired infections. *Antimicrobial Agents and Chemotherapy*, *61*(3), e01709–e01716. 27993852 10.1128/AAC.01709-16 PMC5328522

[CIT0037] Ture, Z., Güner, R., & Alp, E. (2023). Antimicrobial stewardship in the intensive care unit. *Journal of Intensive Medicine*, *3*(03), 244–253. 37533805 10.1016/j.jointm.2022.10.001 PMC10391567

[CIT0038] Udy, A. A., Roberts, J. A., & Lipman, J. (2013). Clinical implications of antibiotic pharmacokinetic principles in the critically ill. *Intensive Care Medicine*, *39*, 2070–2082. 24045886 10.1007/s00134-013-3088-4

[CIT0039] Varghese, J. M., Roberts, J. A., & Lipman, J. (2011). Antimicrobial pharmacokinetic and pharmacodynamic issues in the critically ill with severe sepsis and septic shock. *Critical Care Clinics*, *27*(1), 19–34. 21144984 10.1016/j.ccc.2010.09.006

