# Supplementary material for: The impact of pharmacist-led education and prospective audit and feedback on antibiotic dose optimization within medical intensive care units in Thailand: a retrospective study
Source: J Pharm Policy Pract. 2025 Feb 28;18(1):2467456. doi: 10.1080/20523211.2025.2467456 (PMC11873917; doi:10.1080/20523211.2025.2467456)
Supplement: Supplemental Material [file JPPP_A_2467456_SM2466.docx]

**Supplementary data**

The criteria for prescription evaluation are as the below. We evaluated appropriate prescriptions only prescribed by MICU physicians and the first dose administered at the MICU ward.

**For the first dose prescribed by the MICU physician at the MICU ward following as:**

The first dose should be a normal dose and administered within 30-60 mins as **Table 1**

**Table 1** Dose recommendation for the first dose [Ref: LEXIDRUG^®^ application and previous studies] (Jaruratanasirikul et al., 2019; Kanchanasurakit et al., 2020; Rybak et al., 2020)

| Type of antibiotics | Recommend the first dose |
| --- | --- |
| Meropenem | 1-2 g IV drip in 30 min |
| Imipenem/cilastatin | 500-1000 mg IV drip in 30 min |
| Ertapenem | 1g IV drip in 30 min |
| Piperacillin/tazobactam | 4.5 g IV drip in 30 min |
| Sulbactam | 3 g IV drip in 30 min (Jaruratanasirikul et al., 2019) |
| Cefoperazone/sulbactam | 3 g IV drip in 30 min (Jaruratanasirikul et al., 2019) |
| Ampicillin/sulbactam | 3 g IV drip in 30 min (Jaruratanasirikul et al., 2019) |
| Colistin | 300 mg IV drip in 60 min |
| Fosfomycin | 4-8 g IV drip in 60 min (Kanchanasurakit et al., 2020) |
| Amikacin | 15-20 mg/kg IV drip in 30-60 min, IBW |
| Gentamicin | 7-10 mg/kg IV drip in 30-60 min, IBW |
| Vancomycin | - 1. mg/kg IV drip in 2-3 hours (5mg/min), TBW (Rybak et al., 2020) |

IBW: Ideal body weight

TBW: Total body weigh

1. There is no dose adjustment by the renal or hepatic function or RRT in the first 24 hours at the infection’s time. This criterion was evaluated for all antibiotics listed in ASP

**For maintenance dose prescribed** **by MICU physician at MICU ward following as:**

1. After the first 24 hours, adjustment dose by creatinine clearance (CrCl) or renal replacement therapy (RRT): (Reference: LEXIDRUG ® application). For End-Stage Renal Disease (ESRD) patients, we recommended using dosing CrCl < 10 ml/min. This criterion was evaluated for all non-TDM antibiotics listed in ASP.

- Dose and duration of antibiotic with extended infusion depend on type of RRT, e.g., Continuous veno-venous Hemofiltration (CVVH), Continuous veno-venous hemodialysis (CVVHD), Continuous venovenous hemodiafiltration (CVVHDF), Sustained low-efficiency dialysis (SLED), and hemodialysis (HD) The data are shown in Table 2.

**Table 2** Recommended extended infusion depends on type of renal replacement therapy

| Type of RRT | Extended infusion |
| --- | --- |
| CVVH, CVVHD, and CVVHDF | extended infusion as Table 3 |
| HD and SLED | 1 hr. after HD or SLED on dialysis day |

1. Extended infusion of maintenance doses is required for Meropenem, Imipenem/cilastatin, Piperacillin/tazobactam, Cefoperazone/sulbactam, Ampicillin/sulbactam, Sulbactam [Ref: previous studies] (Jaruratanasirikul et al., 2019; Kanchanasurakit et al., 2020) show as **Table 3**

**Table 3** Recommended extended infusion for non-therapeutic drug monitoring antibiotics

| Type of antibiotics | Recommend extended infusion |
| --- | --- |
| Meropenem | 3 hr. |
| Imipenem/cilastatin | 3 hr. |
| Piperacillin/tazobactam | 3-4 hr. |
| Cefoperazone/sulbactam | 4 hr. (Jaruratanasirikul et al., 2019) |
| Ampicillin/sulbactam | 4 hr. (Jaruratanasirikul et al., 2019) |
| Sulbactam | 4 hr. (Jaruratanasirikul et al., 2019) |
| Fosfomycin | 4 hr. (Kanchanasurakit et al., 2020) |

**For maintenance dose of therapeutic drug monitoring (TDM) antibiotic prescribed by the MICU physician at the MICU ward following as:**

Aminoglycosides (peak and trough concentration) (DOSE)

- 1. trough level 30 mins before 2^nd^-time administration
  2. peak level depends on the duration of administration (30 mins: wait 30 minutes after the end of infusion, 60 mins at the end of infusion)

**Table 4** Target concentration for amikacin and gentamicin (DOSE)

| Target concentration | Amikacin | Gentamicin |
| --- | --- | --- |
| C_peak_ | 40 - 60 mg/dl | 20 - 30 mg/dl |
| C_trough_ | < 4 mg/dl | < 1 mg/dl |
| C_peak_ > MIC: 8-10 | | |

1. Vancomycin: We recommended vancomycin trough level 10-20 mg/dL measuring before the 4^th^ dose for empirical therapy and waiting for culture results. Still, for HD or SLED patients, we recommended measuring vancomycin trough level before the next HD or SLED session.

**Table 5** Target vancomycin depends on type of infection (Habib et al., 2015; Rybak et al., 2020)

| Type of infection | Target vancomycin level |
| --- | --- |
| MRSA infection; MIC < 1 ug/ml | AUC/MIC = 400 – 600 mg*h/L |
| UTIs, SSTIs | C_trough_ 10-15 mg/dl |
| CNS infection, endocarditis, osteomyelitis, pneumonia, septic shock | C_trough_ 15-20 mg/dl |
| *Staphylococcus aureus* endocarditis | C_trough_ 15-20 mg/dl andAUC/MIC < 400mg*h/L |
| C_trough_<10 mg/dl induced resistance for vancomycin and C_peak_ >40 mg/dl are at risk of nephrotoxicity | |

MRSA: Methicillin-Resistant *Staphylococcus aureus* (MRSA)

UTIs: Urinary tract infections

SSTIs: Skin and soft tissue infections

CNS infection: Central nervous system infection

- **Antimicrobial studies are references in our study**.
  1. **Meropenem, Imipenem/cilastatin, and Piperacillin/tazobactam**

We recommended carbapenems (except Ertapenem), and Piperacillin/tazobactam were administered by extended infusion in maintenance dose. A randomized controlled trial compared the administration of beta-lactam antibiotics between continuous infusion versus intermittent bolus resulted in continuous infusion with higher clinical cure rates (p-value = 0.011) and higher median ventilator-free days (p-value<0.043) than intermittent bolus (Abdul-Aziz et al., 2016).

Systematic Review and Meta-analysis showed better pharmacokinetic and pharmacodynamic (PK/PD) properties for extended or continuous infusions of carbapenems and piperacillin/tazobactam. Mortality was lower among patients who received extended or continuous infusions of a carbapenem or piperacillin/tazobactam than those who received short-term RR = 0.59 (95% CI; 0.41-0.83) (Falagas et al., 2013).

- 1. **Cefoperazone/sulbactam, Ampicillin/sulbactam and Sulbactam**

We recommended sulbactam as dose adjustment based on the minimum inhibitory concentration (MIC) of multidrug-resistant (MDR) *Acinetobacter baumannii* in ventilator-associated pneumonia. A pharmacokinetic study on Thai critically ill patients infected with MDR *Acinetobacter baumannii* in VAP found achieving a probability of target (PTA) ≥90% requires a high dose and extended infusion (4 hours) combination of sulbactam (Jaruratanasirikul et al., 2019). For empirical therapy of carbapenem-resistant *Acinetobacter baumannii* (CR-AB), we recommended optimal sulbactam of 12 g/day and 4 hours of infusion to cover MIC 96 μg/mL (Saelim et al., 2018).

- 1. **Colistin**

We recommend that the first dose of colistin should be 300 mg CBA (9 million IU) infused for 60 mins followed the dose recommended by IDSA Colistin in Critically Ill Patients 2017 to achieve colistin concentration at steady state of 2 mg/L in critically ill patients faster than the none-loading dose (Grégoire et al., 2014; Nation et al., 2017).

- 1. **Fosfomycin**

We recommended using optimal fosfomycin dose against Carbapenem-Resistant Enterobacteriaceae based on MIC and Monte Carlo simulation study. For Thai critically ill patients, fosfomycin ranging from 16 to 24 g/day achieves the pharmacokinetic/pharmacodynamic (PK/PD) target. Eleven of the twelve patients who received recommended fosfomycin had bacterial eradication. The microbiological cure rate was 91%, and 2 patients died despite having negative cultures for CRE. The most commonly observed adverse drug reactions were hypernatremia (3 cases) and hypokalemia (3 cases), and acute kidney injury (3 cases) (Kanchanasurakit et al., 2020).

- 1. **Amikacin and Gentamicin**

We recommended administering once-daily regimens. In randomized clinical trials, a once-daily regimen versus multiple daily dosing regimens have been compared for amikacin, netilmicin, and gentamicin indicated once-daily has significant clinical efficacy (89.5 % vs. 84.7 %, p-value < 0.001) as well as bacteriological efficacy (88.6 % vs. 83.4 %, p-value < 0.01) There also were no differences for toxicity (Blaser & König, 1995).

We recommended that all patients who received aminoglycosides performed therapeutic drug monitoring (TDM) followed by Stanford Health Care Aminoglycoside Dosing Guideline (DOSE).

- 1. **Vancomycin**

We recommended a vancomycin loading dose of 20-35 mg/kg. The guideline 2020 suggested that loading doses rapidly achieve targeted level of vancomycin and decrease the risk of subtherapeutic concentrations during the first days of therapy, especially in critically ill patients (Rybak et al., 2020). The prospective observational study by Hodiamont et.al. found that vancomycin loading dose of 25 mg/kg can reach the PK/PD target attainment in the first 24 h in critically ill patients achieving AUC_0–24_ >400 mgh/L increased significantly from 53.8% to 88.0% (p-value=0.0006) (Hodiamont et al., 2021).

We recommended that all patients who received vancomycin perform therapeutic drug monitoring (TDM) which was followed by the Vancomycin guideline 2020 and ESC Guidelines on Infective Endocarditis 2015 (Rybak et al., 2020),(Habib et al., 2015).

- **Acute Physiology and Chronic Health Evaluation II (APACHE II) score** is a severity score and mortality estimation tool developed from a large sample of ICU patients in the United States, created by William Knaus, MD, is a Professor Emeritus of The University of Virginia School of Medicine and a member of The National Academy of Medicine. The APACHE II score comprises 12 physiological variables and 2 disease-related variables (Knaus et al., 1985).

| **APACHE II score** | **Non-operative mortality** | **Operative mortality** |
| --- | --- | --- |
| 0 – 4 | 4 | 1 |
| 5 – 9 | 8 | 3 |
| 10 – 14 | 15 | 7 |
| 15 – 19 | 25 | 12 |
| 20 – 24 | 40 | 30 |
| 25 – 29 | 55 | 35 |
| 30 – 34 | 73 | 73 |
| > 34 | 85 | 88 |

- **Defined Daily Dose (DDD)** is the assumed average maintenance dose per day for a drug used for its main indication in adults, followed World Health Organization (WHO) (Organization, 2020). We calculate DDD in each of the antibiotics monthly.

| Type of antibiotics | Defined Daily Dose (grams) |
| --- | --- |
| Meropenem | 3 |
| Imipenem/cilastatin | 2 |
| Ertapenem | 1 |
| Piperacillin/tazobactam | 14 |
| Sulbactam | 1 (of sulbactam) |
| Cefoperazone/sulbactam | 1 (of sulbactam) |
| Ampicillin/sulbactam | 1 (of sulbactam) |
| Colistin | 9 million unit |
| Fosfomycin | 8 |
| Amikacin | 1 |
| Gentamicin | 0.24 |
| Vancomycin | 2 |

**DDD/1,000 patient-days**

Data about the DDD/1,000 patient-days in each antibiotic is shown below in detail. The dark grey line represents pre-ASP, and the light grey line represents post-ASP. The differences were evaluated by median.

|  |  |
| --- | --- |
| Median: 813.94 to 896.42, p-value=0.356 | Median: 21.29 to 7.15, p-value=0.417 |
|  |  |
| Median: No differences | Median: 237.08 to 323.28, p-value=0.299 |
|  |  |
| Median: 171.41 to 186.11, p-value=0.885 | Median: 369.26 to 280.21, p-value=564 |
|  |  |
| Median: 67.96 to 51.77, p-value=0.954 | Median: 303.01 to 485.04, p-value=0.524 |
|  |  |
| Median: 122.41 to 94.27, p-value=0.488 | Median: 59.16 to 20.26, p-value=0.245 |
|  |  |
| Median: 20.00 to 10.48, p-value=0.65 | Median: 1.54 to 0, p-value=0.753 |

**DOT/1,000 patient-days**

Data about the DOT/1,000 patient-days in each antibiotic is shown below in detail. The dark grey line represents pre-ASP, and the light grey line represents post-ASP. The differences were evaluated by median.

|  |  |
| --- | --- |
| Median: 684.86 to 1,580.96, p-value=0.038 | Median: 22.39 to 0, p-value=0.039 |
|  |  |
| Median: No difference, p-value=0.684 | Median: 197.19 to 300.47, p-value=0.248 |
|  |  |
| Median: 127.58 to 248.83, p-value=0.106 | Median: 257.50 to 181.24, p-value=0.106 |
|  |  |
| Median: 56.79 to 24.76, p-value=0.008 | Median: 62.43 to 34.53, p-value=0.562 |
|  |  |
| Median: 32.66 to 8.87, p-value=0.129 | Median: 43.14 to 0, p-value=0.006 |
|  |  |
| Median: No difference, p-value=0.859 | Median: No difference, p-value=0.970 |

**References**

Abdul-Aziz, M. H., Sulaiman, H., Mat-Nor, M.-B., Rai, V., Wong, K. K., Hasan, M. S., Abd Rahman, A. N., Jamal, J. A., Wallis, S. C., Lipman, J., Staatz, C. E., & Roberts, J. A. (2016). Beta-Lactam Infusion in Severe Sepsis (BLISS): a prospective, two-centre, open-labelled randomised controlled trial of continuous versus intermittent beta-lactam infusion in critically ill patients with severe sepsis. *Intensive Care Medicine*, *42*(10), 1535-1545. <https://doi.org/10.1007/s00134-015-4188-0>

Blaser, J., & König, C. (1995). Once-daily dosing of aminoglycosides. *European Journal of Clinical Microbiology and Infectious Diseases*, *14*(12), 1029-1038.

DOSE, I. D. Stanford Health Care Aminoglycoside Dosing Guideline.

Falagas, M. E., Tansarli, G. S., Ikawa, K., & Vardakas, K. Z. (2013). Clinical outcomes with extended or continuous versus short-term intravenous infusion of carbapenems and piperacillin/tazobactam: a systematic review and meta-analysis. *Clinical infectious diseases*, *56*(2), 272-282.

Grégoire, N., Mimoz, O., Mégarbane, B., Comets, E., Chatelier, D., Lasocki, S., Gauzit, R., Balayn, D., Gobin, P., & Marchand, S. (2014). New colistin population pharmacokinetic data in critically ill patients suggesting an alternative loading dose rationale. *Antimicrobial agents and chemotherapy*, *58*(12), 7324-7330.

Habib, G., Lancellotti, P., Antunes, M. J., Bongiorni, M. G., Casalta, J.-P., Del Zotti, F., Dulgheru, R., El Khoury, G., Erba, P. A., Iung, B., Miro, J. M., Mulder, B. J., Plonska-Gosciniak, E., Price, S., Roos-Hesselink, J., Snygg-Martin, U., Thuny, F., Tornos Mas, P., Vilacosta, I., Zamorano, J. L., & Group, E. S. D. (2015). 2015 ESC Guidelines for the management of infective endocarditis: The Task Force for the Management of Infective Endocarditis of the European Society of Cardiology (ESC)Endorsed by: European Association for Cardio-Thoracic Surgery (EACTS), the European Association of Nuclear Medicine (EANM). *European Heart Journal*, *36*(44), 3075-3128. <https://doi.org/10.1093/eurheartj/ehv319>

Hodiamont, C., Juffermans, N., Berends, S., van Vessem, D., Hakkens, N., Mathôt, R., de Jong, M., & van Hest, R. (2021). Impact of a vancomycin loading dose on the achievement of target vancomycin exposure in the first 24 h and on the accompanying risk of nephrotoxicity in critically ill patients. *Journal of antimicrobial chemotherapy*.

Jaruratanasirikul, S., Nitchot, W., Wongpoowarak, W., Samaeng, M., & Nawakitrangsan, M. (2019). Population pharmacokinetics and Monte Carlo simulations of sulbactam to optimize dosage regimens in patients with ventilator-associated pneumonia caused by Acinetobacter baumannii. *European Journal of Pharmaceutical Sciences*, *136*, 104940.

Kanchanasurakit, S., Santimaleeworagun, W., McPherson III, C. E., Piriyachananusorn, N., Boonsong, B., Katwilat, P., & Saokaew, S. (2020). Fosfomycin dosing regimens based on monte carlo simulation for treated carbapenem-resistant Enterobacteriaceae Infection. *Infection & chemotherapy*, *52*(4), 516.

Knaus, W. A., Draper, E. A., Wagner, D. P., & Zimmerman, J. E. (1985). APACHE II: a severity of disease classification system. *Critical care medicine*, *13*(10), 818-829.

Nation, R. L., Garonzik, S. M., Thamlikitkul, V., Giamarellos-Bourboulis, E. J., Forrest, A., Paterson, D. L., Li, J., & Silveira, F. P. (2017). Dosing guidance for intravenous colistin in critically ill patients. *Clinical infectious diseases*, *64*(5), 565-571.

Organization, W. H. (2020). *Defined Daily Dose (DDD)*. Retrieved 27 November from <https://www.who.int/toolkits/atc-ddd-toolkit/about-ddd>

Rybak, M. J., Le, J., Lodise, T. P., Levine, D. P., Bradley, J. S., Liu, C., Mueller, B. A., Pai, M. P., Wong-Beringer, A., Rotschafer, J. C., Rodvold, K. A., Maples, H. D., & Lomaestro, B. M. (2020). Therapeutic monitoring of vancomycin for serious methicillin-resistant Staphylococcus aureus infections: A revised consensus guideline and review by the American Society of Health-System Pharmacists, the Infectious Diseases Society of America, the Pediatric Infectious Diseases Society, and the Society of Infectious Diseases Pharmacists. *American Journal of Health-System Pharmacy*, *77*(11), 835-864. <https://doi.org/10.1093/ajhp/zxaa036>

Saelim, W., Santimaleeworagun, W., Thunyaharn, S., Changpradub, D., & Juntanawiwat, P. (2018). Pharmacodynamic profiling of optimal sulbactam regimens against carbapenem-resistant Acinetobacter baumannii for critically ill patients. *Asian Pacific Journal of Tropical Biomedicine*, *8*(1), 14.
